# Don’t forget the world outside the lab – a comment on “Illusions of Understanding in the Sciences”

**DOI:** 10.1007/s42113-026-00327-2

**Published:** 2026-09-15

**Authors:** Dominik Bachmann, Leendert van Maanen, Gijs A. Holleman, Christian P. Janssen

**Affiliations:** 1 Institute for Logic, Language and Computation, University of Amsterdam, Amsterdam, The Netherlands; 2 Department of Experimental Psychology and Helmholtz Institute, Utrecht University, Utrecht, The Netherlands; 3 Department of Cognitive Neuropsychology, Tilburg University, Tilburg, The Netherlands

## Abstract

The article “Illusions of Understanding in the Sciences” by Shiffrin et al. (2026) describes ways in which scientists can mislead themselves and others about the extent to which they understand the (causal underpinnings of) phenomena they research. In this reply, we highlight how such “illusions of understanding” can arise from researchers overestimating the extent to which their results generalize beyond curated lab conditions. Since experimental designs are informed by researchers’ theoretical lenses and methodological preferences, we argue that interdisciplinary work (which exposes one to different lenses) on more applied problems (which exposes one to less idealized conditions) can reveal such illusions.

## Response

Shiffrin et al. (2026), hereafter “SSK”, describe several ways in which scientists regularly and inadvertently mislead themselves and others about the extent to which they understand the (causal underpinnings of) phenomena they research. We appreciate the authors’ contribution in compiling an extensive list of reasons for why scientists should be more epistemologically humble. With this reply, we aim to highlight one additional cause for several of their illusions: When designing experiments, researchers consider idealized versions of their phenomena of interest – either because they judge other factors to be (largely) causally irrelevant (Aristotelian idealization; Frigg & Hartmann, 2006) or because idealization makes causal effects easier to isolate (Galilean idealization; see e.g., Weisberg, 2007).

While such idealizations keep experiments practically feasible, what researchers choose to idealize away is colored by their theoretical lenses and methodological preferences. For example, when studying driving safety with semi-automated vehicles, cognitive scientists might focus on mental resources (van der Heiden et al., 2022) or individual differences in reaction speeds of drivers (Bachmann & van Maanen, 2024), but idealize away factors outside drivers – like the physical properties of vehicles or roads. By contrast, engineers might attend closely to these physical properties but idealize away cognitive aspects.^1^

But, as SSK (2026) note, researchers “overestimate the causal effect of whatever variable [they] considered”. Evidence for one’s pet issue being a partial cause ought not to be confused with evidence for it being a relevant cause in the applied setting one abstracted from. This is the case for at least two reasons: Firstly, the partial cause one considered could have a genuine effect in the applied setting, but one that is overshadowed by the effects of other causes. For example, although there is substantial experimental work on how quickly drivers of semi-automated vehicles can take control of their vehicle when alerted (Zhang et al., 2019), in more applied settings, such differences might be overshadowed by whether driving or a non-driving task is the driver’s priority (Janssen et al., 2019).

Secondly, the effects of partial causes one considered could change, once they are no longer experimentally isolated. One’s causes can have (higher-order) interaction effects with other causes which are hidden whenever those other causes are experimentally controlled for. As a hypothetical illustration, a driving simulator experiment might find that more frequent side mirror gazes reduce crash risks. However, such a monotonic “the more mirror gazes, the safer” relationship may not hold in daily commutes, when drivers must also allocate visual attention to other sources of information relevant to safe driving (e.g., traffic signs).

Even when a lab effect is robust, claiming that it operates in applied settings – for example, in a discussion section that speculates about the “practical relevance” of one’s finding – can stretch the evidence. Controlled experiments can tell one that one’s suspected causes could have effects in practice, not that they necessarily do. To adapt a metaphor from Peterson (2025), experiments (especially on complex agents, like humans) are often inadvertently like gardening: Observing that a seed grows in a particular curated environment often tells one more about that environment (e.g., in a particular simulated driving environment, mirror gazes are important) than about intrinsic properties of the seed (e.g., the driver) itself. Generalizations of findings beyond the conditions that produced them are therefore frequently misleading, and researchers should consider and subsequently communicate constraints on the generality of their claims (see also, e.g., Brunswik, 1956; Holleman et al., 2020; Simons et al., 2017).

### A way forward

In our view, several of the issues we raised may be mitigated by tackling (applied) problems multi- or interdisciplinarily, and by involving stakeholders from the domain of practice one wants to generalize to.

Working with researchers from other scientific disciplines is beneficial, because different disciplines apply different lenses and defaults for what to idealize away. Such collaborations can be intellectually humbling and pull one away from one’s habitual approaches to a task: Maintaining SSK’s “illusion of explanatory completeness” for a phenomenon is difficult, if other researchers provide highly differing causal accounts of it (see e.g., Nicenboim et al., 2025).

Tackling more applied problems is beneficial, because it helps one distinguish plausible from practically relevant causes. If one’s pet partial cause has negligible effects in practice, models emphasizing it will poorly explain phenomena in such contexts. Finally, observing and interviewing stakeholders (e.g., workers specialized in the domain one wishes to address) is beneficial because it helps refine theories and experiments (see e.g., Stuut et al., 2025) and can reveal overlooked factors with large(r) effects.

### Concluding thoughts

Many of the illusions of understanding presented by SSK (2026) stem from inherent cognitive limitations of humans (e.g., some stem from forgetting). The practical question to address is hence not “how can we prevent these illusions on an individual level?” but “how can we set up a scientific system that produces good outcomes, despite such (cognitive) limitations of individual researchers?”.

In our view, a promising approach is incentivizing and institutionally supporting interdisciplinary and transdisciplinary research on applied problems. After all, exposure to perspectives from other scientific disciplines, to domain experts, and to predictive limits of one’s theories in application lay bare several “illusions of understanding” that stem from one’s idealizing assumptions.

Of course, this approach is no panacea. Applied and interdisciplinary research comes with unique challenges (outlined e.g., in Holleman et al., 2024) like a lack of a shared vocabulary (e.g., Janssen et al., 2020) – which likely exacerbates SSK’s illusions from miscommunication – and constraints that arise from involving non-academic stakeholders (e.g., needing to deliver a product). But we do believe such trade-offs are often worth it.

## Data Availability

No datasets were generated or analysed during the current study.

## Footnotes

1 Parenthetically, such narrow foci can self-reinforce: An engineer who focuses on road and vehicle design likely devises studies that can speak to these but not to other factors. If they find supportive evidence, that will reinforce their initial idealization choices. Such a feedback loop can cause illusions of understanding like SSK’s “illusion of explanatory completeness”.
